# Rescue and characterization of PCV4 infectious clones: pathogenesis and immune response in piglets

**DOI:** 10.3389/fmicb.2024.1443119

**Published:** 2024-07-29

**Authors:** Lan Chen, Li-Shuang Deng, Tong Xu, Si-Yuan Lai, Yan-Ru Ai, Ling Zhu, Zhi-Wen Xu

**Affiliations:** ^1^College of Veterinary Medicine, Sichuan Agricultural University, Chengdu, China; ^2^Sichuan Key Laboratory of Animal Epidemic Disease and Human Health, College of Veterinary Medicine, Sichuan Agricultural University, Chengdu, China

**Keywords:** porcine circovirus 4 (PCV4), pathogenicity, rescue, immune response, infection model

## Abstract

Porcine circovirus 4 (PCV4) was first identified in 2019, categorized within the genus *Circovirus* in the family *Circoviridae*. To date, the virus has not been isolated from clinical samples. Meanwhile, many aspects of the biology and pathogenic mechanisms of PCV4 infection remain unknown. In this study, PCV4 was successfully rescued from an infectious clone. We utilized a PCV4 virus stock derived from this infectious clone to intranasally inoculate 4-week-old specific-pathogen-free piglets to evaluate PCV4 pathogenesis. The rescued PCV4 was capable of replicating in both PK-15 cells and piglets, with the virus detectable in nearly all collected samples from the challenge groups. Pathological lesions and PCV4-specific antigens were observed in various tissues and organs, including the lungs, kidneys, lymph nodes, spleen, and liver, in the inoculated piglets. Additionally, the levels of pro-inflammatory cytokines in the serum of the PCV4-inoculated group were significantly elevated compared to the control group, indicating that the induced inflammatory response may contribute to tissue damage associated with PCV4 infection. These findings offer new insights into the pathogenesis and inflammatory responses associated with PCV4-related diseases.

## 1 Introduction

Porcine circovirus (PCV) belongs to the genus *Circovirus* in the family Circoviridae. Prior to 2019, only three genotypes, namely PCV1, PCV2, and PCV3, had been documented worldwide (Allan et al., 1998; Hamel et al., 1998; Rodríguez-Arrioja et al., 2000; Lekcharoensuk et al., 2004; Phipps et al., 2007; Krakowka et al., 2008). PCV1 was a non-pathogenic viral particle (Gagnon et al., 2010; Ma et al., 2011; Saha et al., 2011). PCV2 is the predominant pathogen responsible for PCVAD, characterized by immunosuppression leading to clinical signs (Krakowka et al., 2008; Becskei et al., 2010; Doster et al., 2010; Henriksson et al., 2011; Resendes et al., 2011). In October 2016, PCV3 was detected in the USA (Wang et al., 2020). Subsequently, PCV3 has been detected in countries such as Poland, Japan, and South Korea (Kwon et al., 2017; Stadejek et al., 2017; Hayashi et al., 2018; Deng et al., 2023). In 2019, PCV4, was identified in farmed pigs, in Hunan Province, China (Zhang et al., 2020). It was also reported in several provinces and cities in China and South Korea (Tian et al., 2021; Hu et al., 2022; Wang et al., 2022; Chen et al., 2023; Fu et al., 2024). Extensive research has indicated that PCV2 and PCV3 cause a series of serious diseases and syndromes in swine farms, posing a threat to the health of pigs and the economic efficiency of the farming industry (Mora-Díaz et al., 2020; Yang et al., 2022). However, the pathogenicity and harmfulness of PCV4 have not been fully clarified. As a newly discovered virus, the inability to isolate PCV4 from clinical samples significantly hampers the assessment of its pathogenicity, the development of effective prevention and control strategies, and the creation of vaccines against it.

Infectious clones are typically created by inserting the viral genome into a plasmid vector, which can then be transcribed into RNA or directly used to produce infectious virus particles in host cells (Fenaux et al., 2002; Park et al., 2016; Jiang et al., 2019; Niu et al., 2022; Shakir et al., 2023). This approach allows detailed investigation of viral functions, including gene expression, replication mechanisms, and host interactions. For viruses that are difficult to isolate, we often employ the method of infectious clones. In 2002, Fenaux et al. (2002) reported the first construction and use of an infectious molecular DNA clone of PCV2. This molecular clone was used for direct *in vivo* transfection of pigs to characterize the disease and associated pathological lesions of PCV2 infection. Jiang et al. (2019) have recently reported that PDNS-like disease can be reproduced in pigs infected with a cloned PCV3 virus in 2019.

In this study, we utilized the predominantly endemic strain in the Southwest of China to construct the pSK recombinant plasmid and we developed the rescued virus expression vector pSK-PCV4 (Xu et al., 2022c). Pathological lesions and PCV4-specific antigens were detected in various tissues and organs, including the lungs, kidneys, lymph nodes, spleen, and liver. Additionally, there was a notable increase in pro-inflammatory cytokines and chemokines in the serum. These results contribute to the assessment of PCV4 infectivity and pathogenicity, providing valuable insights for the prevention, treatment, and vaccine development for related diseases.

## 2 Materials and methods

### 2.1 Plasmid construction and virus rescuing

The full-length genome of PCV4 was synthesized based on the sequence of PCV4/SC-GA2022ABTC (OP497960.1) published by our laboratory. A molecular DNA clone of the PCV4 genome, flanked by Sac I and Hind III restriction enzyme sites at the 5′ and 3′ ends respectively, was inserted into the pBluescript SK+ vector, which had been predigested with the same enzymes to create the recombinant plasmid pSK-PCV4. Sequencing of the recombinant plasmid confirmed the absence of errors introduced during PCR amplification.

When the cells reached approximately 70% confluency, 250 ng of recombinant plasmid was transfected into the cells using Lipofectamine 3,000 reagent (Thermo Fisher Scientific, Waltham, MA, United States). The cells were additionally treated with 300 mM D-glucosamine for 30 min at 24 h after transfection. Then, the old medium was discarded and the cells was incubated with DMEM containing 2% FBS.

Seventy-two hours after transfection, the cells were subjected to three successive freeze–thaw cycles and named F0. Inoculate the F0 into freshly cultured PK-15 cells and perform successive passaging within the PK-15 cell line. The supernatants of virus culture medium were collected from generations F0 to F6, respectively. Total viral DNA was extracted from the collected samples using the DNA/RNA Extraction Kit (Vazyme, Nanjing, China). PCV4 genomic copies were quantified using a validated TB Green II-based real-time quantitative PCR (qPCR) assay, following the protocol described before (Xu et al., 2022b).

### 2.2 Identification of rescued PCV4

The generated PCV4 virus stock was then serially passaged in PK15 cells, and the titer of the PCV4 virus stock was determined as described previously for PCV2 and PCV3 titration and expressed as the 50% tissue culture infective dose (TCID_50_)/ml (Fenaux et al., 2002; Jiang et al., 2019). To confirm the virus was successfully rescued, the F6 virus was used to infect PK15 cells, and the cells were subjected to an immunofluorescence assay after 72 h.

Simultaneously, the infected PK15 cells were washed twice with pre-cooled phosphate-buffered saline (PBS, pH7.4) after removing the culture medium. Subsequently, 200 μL of RIPA cell lysis buffer was added to each well of a 6-well plate and incubated on ice for 20 min. Following incubation, all lysed samples were scraped off with a sterile cell spatula and transferred to pre-cooled 1.5 mL centrifuge tubes. The supernatant was then centrifuged at 12,000 rpm at 4°C for 10 min to isolate total cellular proteins. The resulting supernatant constituted the total cellular protein extract. The protein level of the rescued virus was further assessed through western blot analysis of the Cap protein from the F6 generation virus, using a mouse anti-PCV4 Cap polyclonal antibody developed in our laboratory as the primary antibody. For a detailed description of the preparation process, refer to the Supplementary material S1.

### 2.3 Animal experiment

Six 4-week-old specific pathogen-free piglets purchased from Wanjiahao in Meishan, Sichuan, were randomly divided into two groups: control group and PCV4 inoculation group. Each group of three piglets was housed in a separate room and provided with sterile food and water *ad libitum*. Before inoculation, all piglets tested negative for PCV2, PCV3, PRV, CSFV, PEDV, JEV and PRRSV by qPCR or RT-qPCR. After a one-week acclimation period, the PCV4-inoculated group received an nasal injection with 1 × 10^5.93^ TCID_50_/ml of the PCV4, while the control group was similarly injected with PBS. After 28 days, all piglets were humanely euthanized.

### 2.4 Clinical signs

All pigs were weighed daily, and relative weight gain were calculated weekly. Rectal temperatures were recorded daily. Clinical observations were documented each day.

### 2.5 Sample collection

Blood samples were collected from all pigs every 3 days and placed in serum separation tubes. The blood was centrifuged at 2000 rpm for 10 min. The serum was stored at −80°C for future use.

At day 28, necropsies were performed, and samples of the heart, liver, spleen, lungs, kidneys, tonsils, and lymph nodes were collected into separate bags and stored at −80°C.

### 2.6 Assessment of viremia and distribution of PCV4 in various organs

PCV4 viral loads in tissues and sera were measured by qPCR described previously (Xu et al., 2022b). Samples underwent a 10-fold dilution in PBS, were then vortexed at 4°C, and subsequently centrifuged at a speed of 12,000 rpm for a duration of 10 min. The extracted DNA was counted and stored at −80°C. Results were analyzed using Bio-Rad CFX Manager 4.1 (Bio-Rad Laboratories, Shanghai, Co., Ltd.).

### 2.7 HE and IHC of rescued PCV4-inoculated piglets

At the end of the 28-day experiment, gross pathological lesions were observed. The same tissue samples were then fixed in 4% paraformaldehyde, dehydrated through a graded ethanol series, and embedded in paraffin. Subsequently, the samples were sectioned into 4 μm thick slices and stained with hematoxylin and eosin (H&E) for histopathological examination under an optical microscope.

For IHC, Firstly, tissue sections are deparaffinized in xylene and rehydrated through graded alcohols to water. Antigen retrieval is performed by heating the sections in a citrate buffer solution. The slides are then cooled and rinsed with PBS. To block endogenous peroxidase activity, the sections are incubated with hydrogen peroxide. The mouse anti-PCV4 Cap polyclonal antibody is then applied to the sections and incubated at 4°C overnight. After rinsing with PBS, the sections are incubated with an HRP-conjugated goat anti-mouse IgG (Bioss, Beijing, Co., Ltd.). Following this, a chromogen such as DAB (Servicebio, Wuhan, Co., Ltd.) is added to visualize the antibody–antigen complex, resulting in a brown precipitate. The stained sections are then examined under a microscope to assess the localization and intensity of the antigen expression.

### 2.8 ELISA of cytokine

The serum levels of IL-1β, IL-6, IL-10, IL-12, IFN-γ, and TNF-α at 0, 7, 14, 21, and 28 days were measured using an ELISA assay (Jianglai Biotechnology, Shanghai, Co., Ltd.).

### 2.9 Statistical analysis

Statistical analysis was performed using GraphPad Prism 9.5.1 (GraphPad Software, San Diego, CA, United States). Data were analyzed using two-way ANOVA followed by *t*-tests to determine statistical significance, with a *p*-value of less than 0.05 considered statistically significant.

## 3 Results

### 3.1 Generation of PCV4 infectious stock

**Figure 1:**
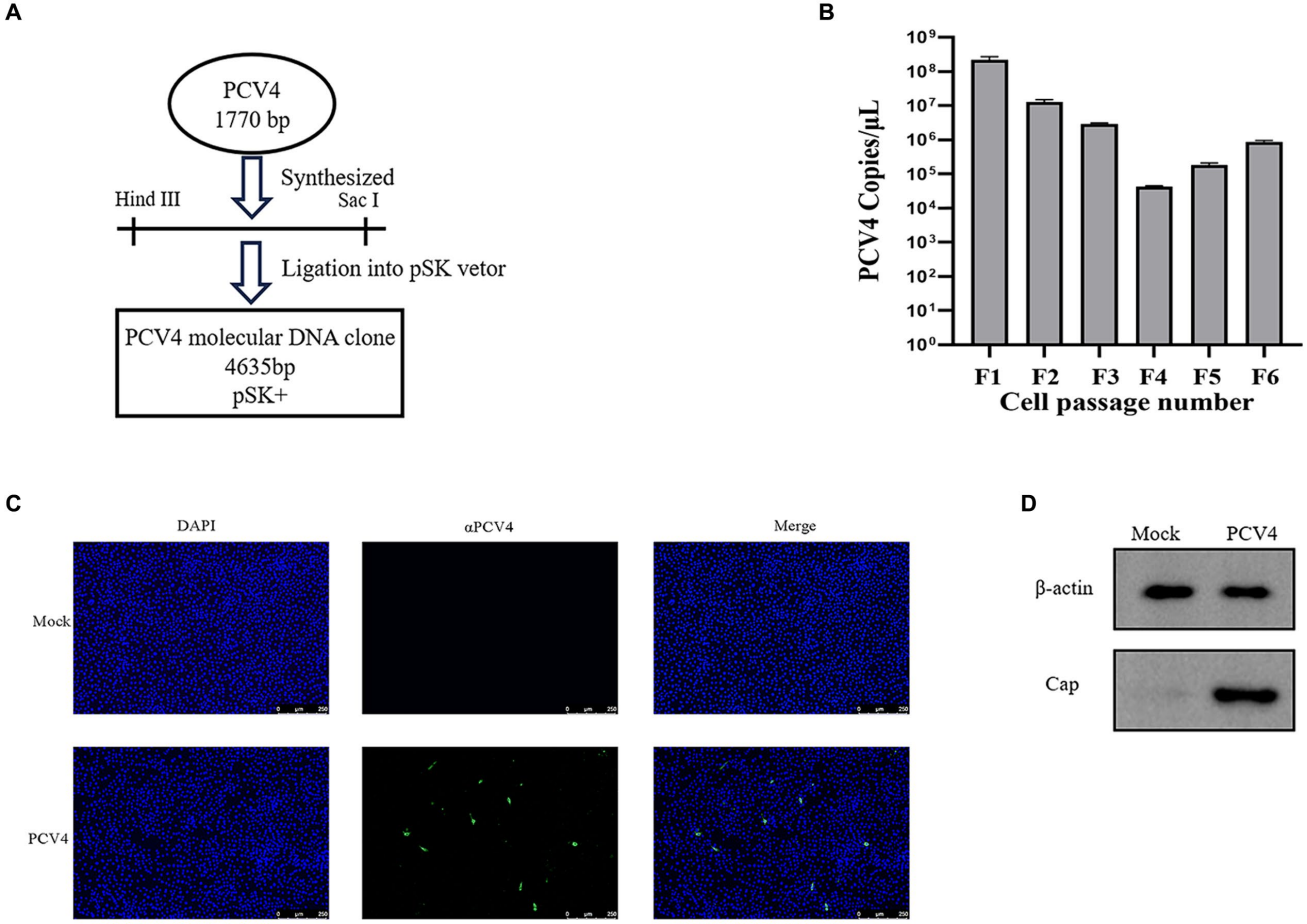
PCV4 infectious clone. **(A)** Construction of an infectious PCV4 molecular DNA clone. **(B)** The copy numbers of each passage. **(C)** Immunofluorescence analysis of PCV4-infected cells. Cells were stained with mouse anti-PCV4 Cap polyclonal antibody (1:100), followed by FITC-labeled goat anti-mouse IgG (green, 1:100). The nuclei were stained with DAPI (blue, 1:500). **(D)** Extract the total protein from the sixth-generation viral fluid for Western blot (WB) analysis.

The full-length genome of PCV4 was synthesized based on the sequence of PCV4/SC-GA2022ABTC (OP497960.1) published by our laboratory. The synthesized PCV4 genome was ligated into the pSK vector to produce the PCV4 clone (Figure 1A). To obtain PCV4 viral particles, PCV4 infectious clones were transfected into PK-15 cells and passaged continuously. The viral load of PCV4 was monitored by qPCR at each passage, confirming the presence of PCV4 genomic DNA in every passage, indicative of viral genome replication within the cells (Figure 1B). Throughout the experimental period, no discernible cytopathic effects (CPE) were observed in transfected PK-15 cells. IFA results revealed that the PCV4 Cap protein within cells infected by the sixth generation of the virus exhibited significant reactivity with mouse anti-PCV4 Cap polyclonal antibodies. Relative to the control group, the widespread distribution of PCV4 within the cells was prominently evident, with a predominance observed in the cytoplasm compared to the nucleus, underscoring the successful rescue of PCV4 and its replication within the cellular environment (Figure 1C). The viral titer of the PCV4 stock after six passages was quantified as 10^5.93^ TCID_50_/ml at 72 h post-infection. Furthermore, Western blot analysis detected a distinct 27 kD PCV4 Cap protein band in cell cultures collected from the sixth generation, contrasting with the absence of bands in the control group, indicative of expression of the viral Cap gene within the cells (Figure 1D).

### 3.2 Clinical signs induced by PCV4 infection

**Figure 2:**
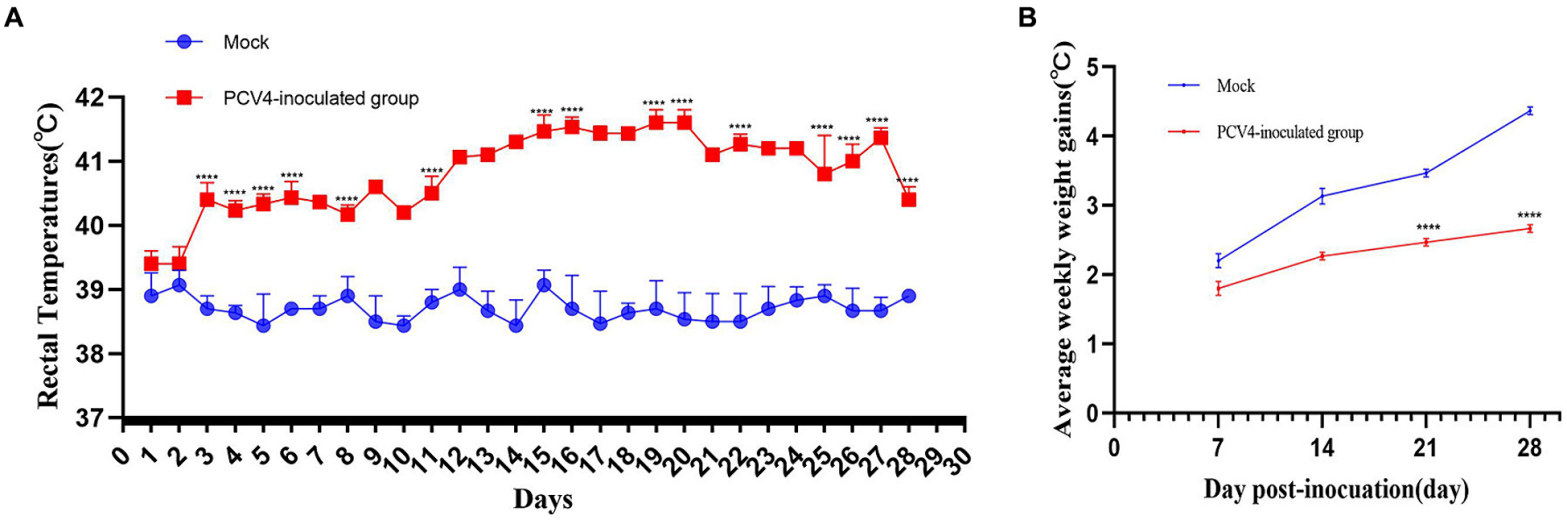
**(A)** Rectal temperature kinetics of the 4-week-old PCV4-inoculated piglets. **(B)** Average weekly weight gains of the 4-week-old PCV4-inoculated piglets following inoculation. * Means *p* < 0.05, ** means *p* < 0.01, *** means *p* < 0.005, **** means *p* < 0.0001 when comparing the PCV4-inoculted group with the control group.

In the PCV4-inoculated group, one of the three piglets first exhibited clinical symptoms such as loss of appetite and diarrhea at 17 days post-infection (dpi). However, no noticeable clinical symptoms were observed in the other piglets inoculated with PCV4. The marked pathological changes in multiple organs of the PCV4-inoculated piglets indicate its pathogenicity in piglets. Rectal temperatures were measured every day. Piglets in the PCV4 inoculated group exhibited varying degrees of elevated body temperature (Figure 2A). Following PCV4 inoculation, weight gain in the PCV4 group piglets decreased after 21 dpi and continued to decline until the end of the experiment (Figure 2B). As expected, the control group showed no significant clinical symptoms.

### 3.3 Assessment of viral loads in sera and organs post-dissection

**Figure 3:**
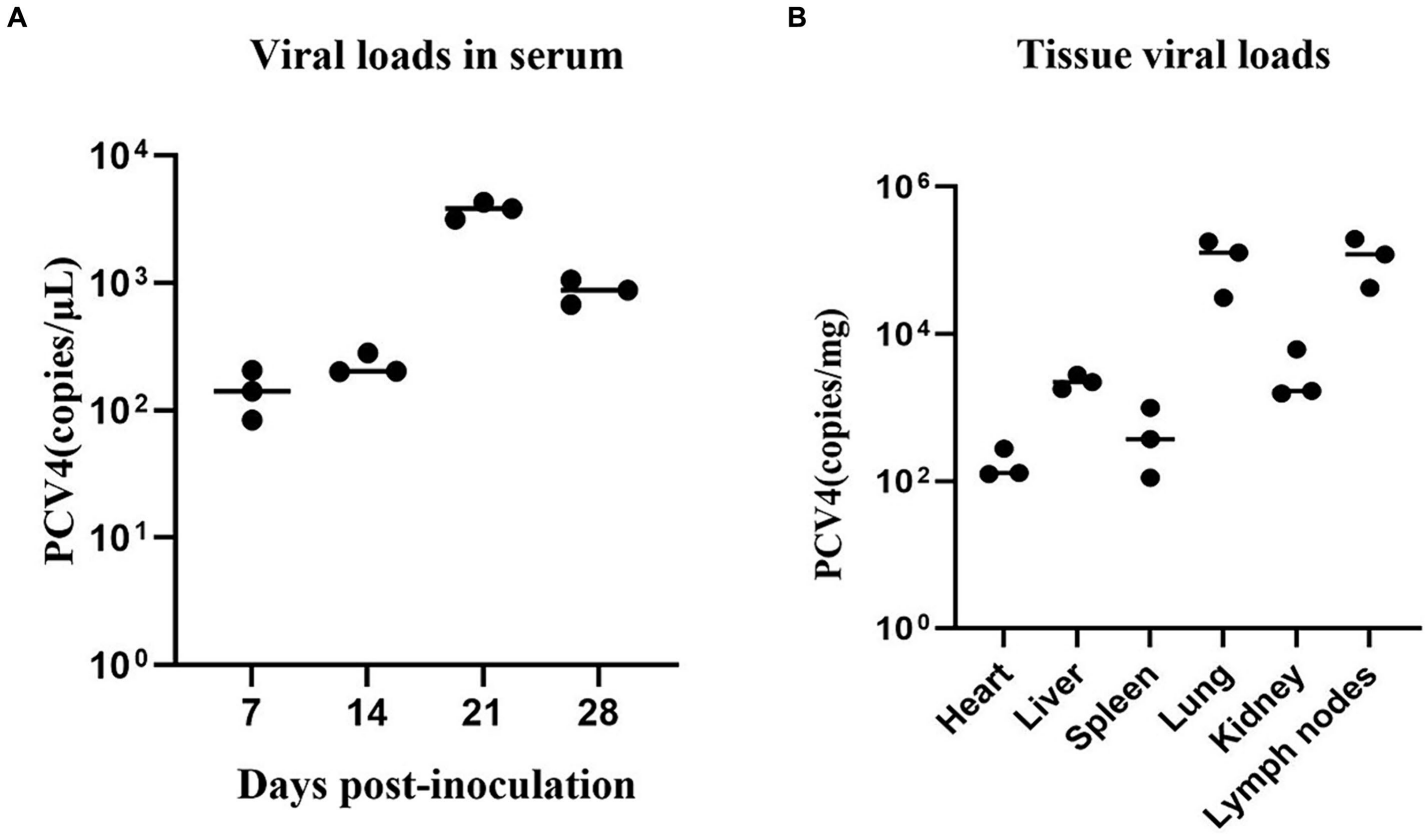
**(A)** PCV4 loads in sera from 4-week-old. PCV4-inoculated piglets were quantitatively assayed by qPCR. **(B)** Viral load of 4-week-old piglets in different tissues. * Means *p* < 0.05, ** means *p* < 0.01, *** means *p* < 0.005, **** means *p* < 0.0001 when comparing the PCV4-inoculted group with the control group.

Throughout the study, all pigs in the control group tested negative for PCV4 in serum samples. In contrast, pigs in the infection group exhibited a significant increase in viremia starting at 7 dpi, peaking at 21 dpi (Figure 3A).

As shown in Figure 3B, PCV4 was detected in the heart, liver, spleen, lungs, kidney, and lymph nodes using qPCR. The detected genomic copies indicated that the lungs and lymph nodes had the highest PCV4 copy numbers. No PCV4 infection was observed in the brain, small intestine, testes, or ovaries.

### 3.4 Histopathological lesions by PCV4 infection

**Figure 4:**
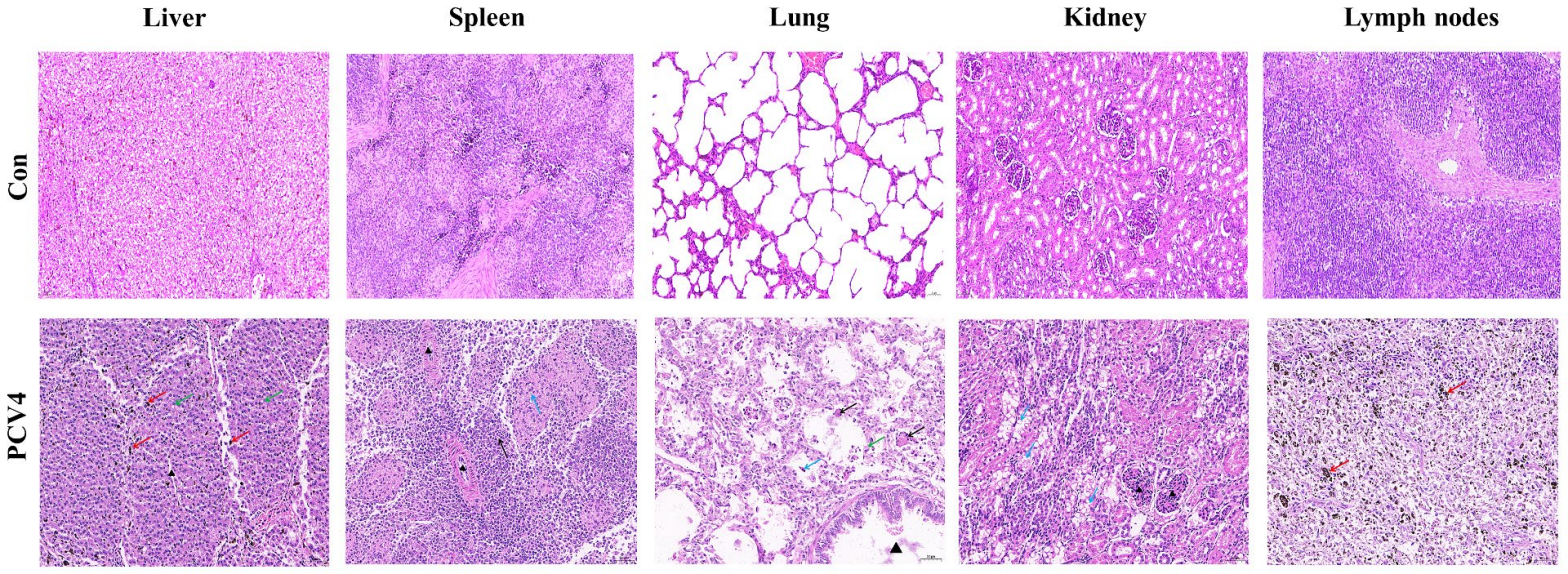
Histopathological lesions of organs in the 4-week-old PCV4-inoculated piglets. Bars, 50 μm.

Histopathological analysis revealed significant changes in the group inoculated with PCV4 Hepatocytes displayed mild vacuolar degeneration, with significant bile stasis evident between hepatocytes and bile ducts. In the spleen, there was a reduction in white pulp cells, accompanied by degeneration and necrosis of reticular cells in the red pulp. Lung tissues exhibited structural damage, characterized by alveolar wall rupture and degeneration, leading to shedding of alveolar epithelial cells into the alveolar cavity. Additionally, alveolar expansion and lymphocyte infiltration were observed. Renal tubules displayed moderate vacuolar degeneration of epithelial cells, along with tubular lumen narrowing and mesangial cell proliferation. Lymph nodes exhibited localized atrophy, with blurred boundaries between the cortex and medulla. The cortical lymphoid follicles were atrophied and reduced in number, with increased lymphocyte apoptosis/necrosis and prominent pigment deposition (Figure 4).

### 3.5 Tissue distribution of PCV4 antigen

**Figure 5:**
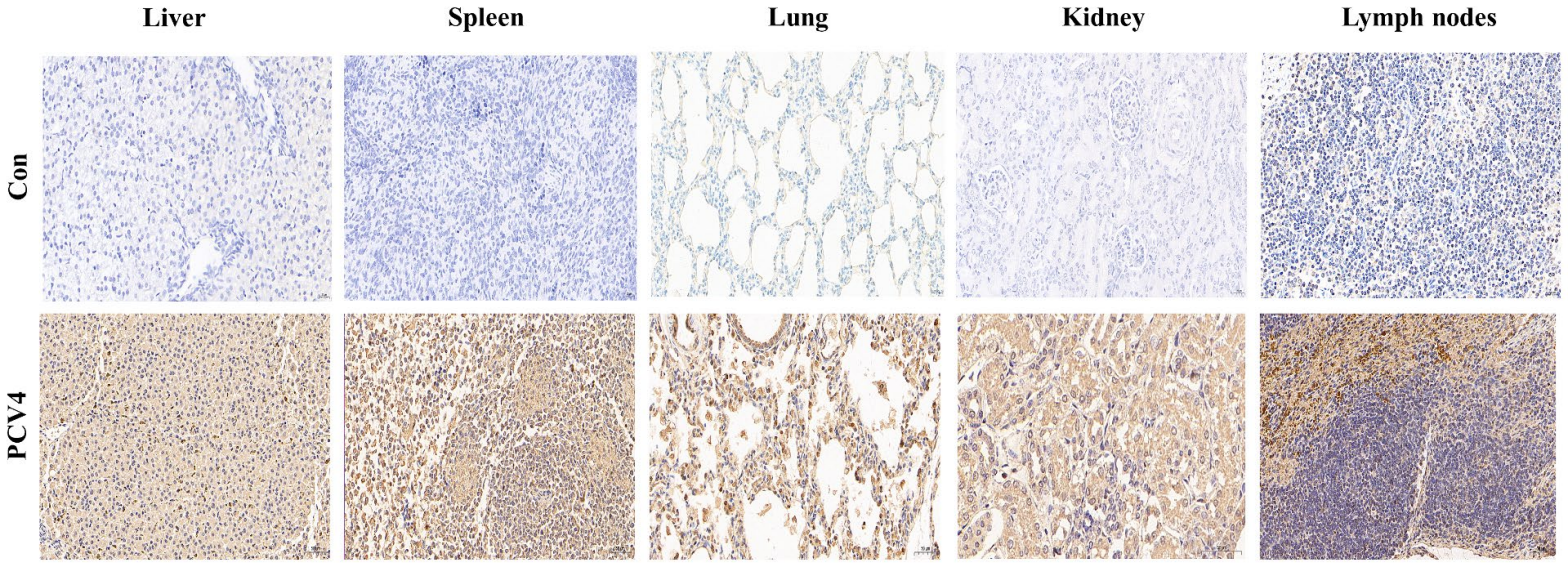
Immunohistochemical staining of organs and tissues of the 4-week-old piglets inoculated with PCV4.

Immunohistochemical staining for PCV4 antigen was performed on the lungs, liver, kidney, spleen, and lymph nodes of piglets. The tissues from control piglets were negative for PCV4 antigen. In the liver, cells within the hepatic sinusoids exhibited a marked positive reaction. In the lungs, both the alveolar walls and the exfoliated cells within the alveolar spaces showed strong positive signals for the PCV4 antigen. In the spleen, the positive reactions were mainly distributed in the red pulp, with a few positive reactions observed in the lymphocytes of the white pulp. In the lymph nodes, clear positive reactions were detected in the cortical necrotic areas and around some local lymphoid follicles. In the kidney, there was a slight positive reaction within the epithelial cells of the renal tubules (Figure 5).

### 3.6 The expression levels of cytokine in PCV4 infected piglets

**Figure 6:**
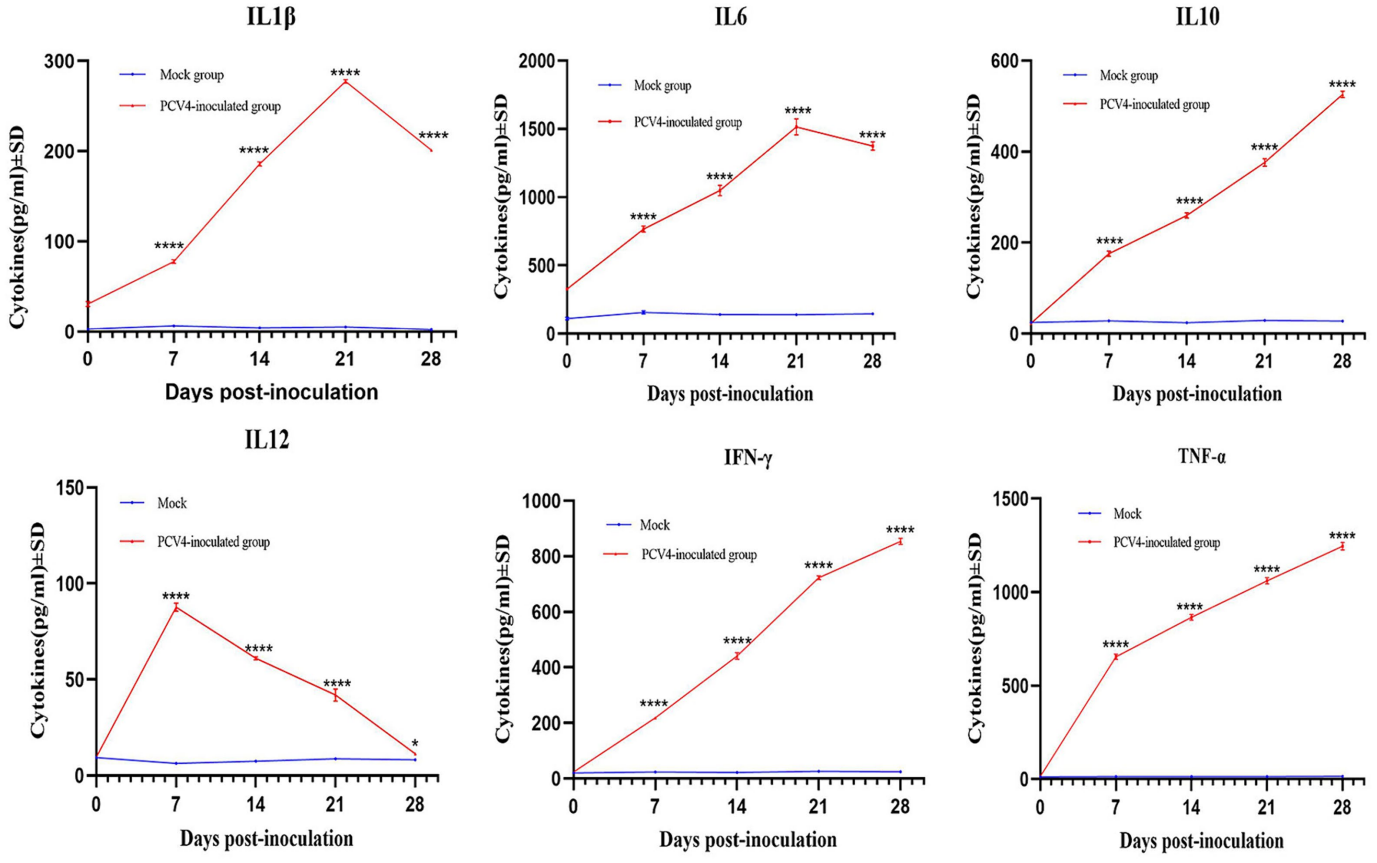
Cytokine/chemokine levels in the sera of the 4-week-old PCV4-inoculated piglets.

To further confirm that PCV4 infection induces inflammatory responses in piglets, we analyzed the expression levels of cytokines in the serum. As shown in Figure 6, compared to the control group, the expression levels of IL-1β, IL-6, and TNF-α in the PCV4-inoculated group continuously increased during the infection period, peaking at 21 days. The expression level of IL-10 and IFN-γ in the PCV4-inoculated group gradually increased throughout the infection period. In contrast, the expression level of IL-12 was low and continuously decreased during the infection period.

## 4 Discussion

PCV4, as a newly virus, which has a genome of 1770 nucleotides and encodes key replication and capsid proteins, has been reported across numerous Chinese provinces and detected in various species, indicating potential cross-species transmission (Tian et al., 2021;Wang et al., 2022; Wu et al., 2022; Xu et al., 2022c; Xu et al., 2023).

Currently, most research on PCV4 is limited to epidemiological investigations and cross-species transmission studies (Hou et al., 2022; Xu et al., 2022c). Although PCV4 was first identified in 2019, retrospective studies indicate that cases of PCV4 infection existed in China as early as 2008. The exact pathogenic mechanisms of PCV4 remain unclear due to the inability to successfully isolate the virus, posing challenges for disease prevention.

Fenaux et al. (2002) constructed an infectious molecular clone of PCV2 and demonstrated its infectivity by directly injecting the cloned PCV2 plasmid DNA into the liver and lymph nodes of pigs. This approach employed *in vitro*-constructed recombinant plasmids and an *in vivo* transfection system to elucidate the structural and functional relationships of PCV2 genes. Through this method, the roles of various regions or genes of PCV2 in viral replication and pathogenesis within the host were elucidated. PCV3, rescued from infectious clones, can induce typical clinical signs in piglets, such as PMWS and PDNS, respectively (Jiang et al., 2019). The clinical signs induced by these rescued viruses closely resemble those observed in field cases, indicating that infectious clones are effective for rescuing PCVs (Fenaux et al., 2002; Jiang et al., 2019). In this study, akin to the methodology employed for PCV2, PCV3 and PCV4 infectious clones, we utilized the PSK vector (Niu et al., 2022). Specifically, we employed the sequence of the SC-GA2022ABTC strain (belonging to PCV4c), which was initially identified in the southwestern region of China and is characterized by a distinctive amino acid sequence (239V for the Rep protein, and 27N, 28R, and 212M for the Cap protein) (Xu et al., 2022c).

We collected supernatants from each viral passage and quantified PCV4 viral load using qPCR. qPCR results indicated high copy numbers in the F1 and F2 generations, potentially attributable to residual transfected plasmid. Meanwhile, IHC and WB results demonstrated that the Cap protein continued to be expressed after multiple passages, confirming the successful rescue of the PCV4 infectious clone strain in this study. Constructing an infectious clone of PCV4 is advantageous as it allows for the generation of a biologically pure and homogeneous infectious virus stock, essential for definitive characterization of the disease and pathological lesions associated with PCV4 infection in piglets.

To assess the pathogenicity of the rescued PCV4 infectious clone strain and its capacity to elicit immune responses, we inoculated intranasally 4-week-old piglets with the rescued virus. The experimental results indicated that viremia was detectable as early as 7 dpi, reaching peak at 21 dpi. Previous studies have shown that Severe lymphoid depletion t in lymphoid tissues are the characteristic histological lesions of the PCV2 and PCV3 (Beach and Meng, 2012; Segalés, 2012). HE staining showed significant lymphocyte infiltration and increased apoptosis/necrosis of lymphocytes in piglets inoculated with the PCV4 infectious clone strain. This suggests that PCV4 infection compromised the pigs’ immune systems, leading to immunosuppression. IHC revealed a substantial presence of PCV4-positive cells across various tissues and organs in the inoculated piglets. These observations suggest that the tissue lesions caused by PCV4 are linked not only to viral replication within the host but also to the virus-induced inflammatory response. Furthermore, PCV4 was detected in several organs, including the heart, liver, spleen, lungs, kidneys, and lymph nodes. This indicates that PCV4 exhibits tissue tropism. The genomic copy numbers detected in different tissues indicated that the lungs and lymph nodes contained the highest PCV4 copy numbers. Previous studies have demonstrated that PCV2 and PCV3 can replicate their viral genomes more efficiently within lymph nodes (Phipps et al., 2007; Krakowka et al., 2008; Mora-Díaz et al., 2020), making these nodes the most common sites for PCV3 investigation and detection. Our research indicates that PCV4 may replicate more effectively in both lymph nodes and lungs.

Pigs infected with PCV4 exhibited milder clinical signs and no piglets died during the study period. This suggests that, under laboratory conditions, the pathogenicity of PCV4 may be relatively low. This is different from the infection of PCV2. Histopathological lesions in multiple tissues and organs similar to those of PMWS were reproduced with the PCV2 molecular DNA clone as well as with the infectious virus prepared *in vitro* from the molecular DNA clone. Besides, piglets that were inoculated with PCV3 alone and been reported in cases of natural infection with the virus presented characteristics of PDNS-like clinical signs and lesions. The clinical symptoms and severity of PCV4-induced disease may be influenced by several factors, including the immune status of the pig population, and co-infection with other porcine pathogens.

Pro-inflammatory cytokines play a crucial role in the development and maintenance of inflammation, potentially leading to multi-organ damage. To confirm that PCV4 infection triggers an inflammatory response, we measured cytokine (IL-1β, IL-6, IFN-γ and TNF-α) expression in pig serum. IL-1β is expressed in various tissues and cells, especially in macrophages within lymphoid organs. IL-6 is not a specific marker for infection, as its levels can increase in response to tissue injury or inflammatory stimuli. TNF-α is a key cytokine involved in inflammation and immune regulation, promoting the production of other cytokines and immune responses. The levels of IL-1β, IL-6, IFN-γ and TNF-α in the PCV4-inoculated group remained elevated throughout the infection period. Same infection as PCV2 and PCV3, upregulation of pro-inflammatory cytokines appears to play an important role in PCV4 pathogenesis (Meng, 2013; Chen et al., 2023).

## 5 Conclusion

PCV4, rescued from infectious clones, demonstrates the ability to replicate both *in vitro* and *in vivo*, leading to mild pathological lesions in piglets. This study substantiates the role of PCV4 in subclinical infections, which are marked by mild multisystemic inflammation and lymphocyte depletion in immune organs. These findings offer new insights into the pathogenesis and inflammatory responses associated with PCV4-related diseases, which are instrumental in developing effective prevention and control strategies against PCV4. Further research is crucial to understand and elucidate the mechanisms of interaction between PCV4 and its host, particularly in terms of immune evasion and pathogenesis.

## Data availability statement

The raw data supporting the conclusions of this article will be made available by the authors, without undue reservation.

## Ethics statement

The animal study was approved by Sichuan Agricultural University Animal Ethical and welfare Committee [license number: 20220283]. The study was conducted in accordance with the local legislation and institutional requirements.

## Author contributions

LC: Writing – review & editing, Writing – original draft. L-SD: Writing – original draft, Validation, Supervision. TX: Writing – original draft, Validation, Supervision. S-YL: Writing – original draft, Conceptualization. Y-RA: Writing – original draft, Methodology. LZ: Writing – original draft, Conceptualization. Z-WX: Writing – review & editing, Writing – original draft, Project administration.
