# Supplementary material for: Rescue and characterization of PCV4 infectious clones: pathogenesis and immune response in piglets
Source: Front Microbiol. 2024 Jul 29;15:1443119. doi: 10.3389/fmicb.2024.1443119 (PMC11317377; doi:10.3389/fmicb.2024.1443119)
Supplement: Supplementary file 1 [file Presentation_1.pdf]

Ten 4-week-old specific pathogen-free (SPF) female Kunming mice were used as experimental animals. High-titer antisera were generated by subcutaneous immunization with purified recombinant protein administered four times. The detailed protocol is as follows:

Initial Immunization: The recombinant protein was diluted to a concentration of 1 mg/mL and emulsified with Freund's adjuvant at a 1:1 ratio. High-speed vortexing was used to achieve proper emulsification, verified when the emulsion floated on water without dispersing upon gentle shaking. Each mouse received 0.2 mL of the emulsion subcutaneously at multiple sites, delivering a total of 100 μg of protein.

Secondary Immunization (Day 14): A second immunization was performed using incomplete Freund's adjuvant. Mice were injected subcutaneously with 0.2 mL of the emulsion at the same dosage.

Tertiary Immunization (Day 21): Seven days after the second immunization, a third injection was administered following the same procedure and dosage as the second immunization.

Quaternary Immunization (Day 28): Seven days after the third immunization, a fourth injection was performed under the same conditions as the previous immunizations.

Serum Collection (Day 35): Seven days following the final immunization, blood was collected via retro-orbital bleeding. Mice were euthanized, and the blood was transferred to 1.5 mL Eppendorf tubes. The samples were allowed to clot at 37°C for 1 hour, then stored at 4°C overnight. After centrifugation at 5000 r/min for 5 minutes, the serum was aliquoted and stored for subsequent antibody efficacy testing.
